# Influence of acetate- vs. lactate-containing fluid bolus therapy on acid-base status, electrolytes, and plasma lactate in dogs

**DOI:** 10.3389/fvets.2022.903091

**Published:** 2022-07-29

**Authors:** Ute Klein-Richers, Annika Heitland, Katrin Hartmann, René Dörfelt

**Affiliations:** ^1^Clinic of Small Animal Medicine, Center for Clinical Veterinary Medicine, LMU Munich, Munich, Germany; ^2^AniCura Tierklinik Haar GmbH, Haar, Germany

**Keywords:** balanced crystalloid solutions, acid-base disorders, hyperlactatemia, metabolic acidosis, canine

## Abstract

**Objective:**

Acetate- and lactate-containing fluids influence the acid-base and electrolyte status. This prospective, randomized, clinical study compared two balanced crystalloid solutions regarding their influence on acid-base status, electrolytes, and lactate values, when given to dogs as a resuscitation bolus of 30 mL/kg.

**Material and methods:**

One hundred client-owned dogs presenting to the emergency service with signs of fluid deficits were randomly assigned to receive an intravenous bolus of 30 mL/kg of either a lactate- (LAC), or an acetate-containing solution (ACET). Before and after the bolus, vital parameters were assessed, and a venous blood gas analysis was performed.

**Results:**

Both solutions performed equally well in decreasing the heart rate (ACET: −10 ± 27 bpm, LAC: −12 ± 30 bpm; *p* = 0.737). The acetate-containing solution caused a significant decrease in plasma lactate levels (*p* = 0.016), anion gap (*p* < 0.001), and potassium (*p* < 0.001), and a significant increase in chloride (*p* < 0.001), and ionized calcium (*p* = 0.014). The lactate-containing solution caused a significant decrease in anion gap (*p* < 0.001), sodium (*p* = 0.016), and potassium (*p* = 0.001), and a significant increase in chloride (*p* < 0.001). ACET causes a stronger decrease in plasma lactate (*p* = 0.015), sodium (*p* = 0.039), potassium (*p* = 0.006), and an increase in chloride (*p* < 0.001), and ionized calcium (*p* = 0.016) compared to LAC.

**Conclusion:**

Both solutions caused mild changes in electrolyte concentrations and had minor influence on acid-base status when used for bolus therapy in dogs with fluid deficits. Further studies are needed to evaluate their influence on acid-base status, lactate, and electrolytes when used in larger volumes and for a longer time span.

## Introduction

Hypovolemia and dehydration are common findings in small animal emergency medicine. Cats and dogs experience fluid losses due to a variety of disorders, such as vomitus, diarrhea, polyuria, or hemorrhage (1). With severe fluid losses, tissue perfusion and oxygen delivery become impaired, forcing cells to rely on anaerobic metabolism to obtain energy. This increases production of lactate and hydrogen ions, leading to metabolic acidosis (1–4). Intravenous fluid therapy helps restore tissue perfusion and aerobic metabolism, thus mitigating acidosis and hyperlactatemia. Isotonic saline (NaCl 0.9%) and balanced replacement fluids are both suitable fluid choices for fluid resuscitation, restoration of blood volume and tissue perfusion, but balanced fluids are considered superior to NaCl 0.9%, due to their more physiological composition. Isotonic saline only contains sodium and chloride in supraphysiological concentrations, and has been shown to induce or exacerbate hyperchloremic metabolic acidosis, which can lead to renal vasoconstriction (5–10). Balanced replacement fluids contain a composition of electrolytes similar to the patients' plasma, and additionally organic anions, such as lactate, malate, or acetate, as buffering substances (8, 9, 11, 12). When these anions are metabolized, protons are consumed, which facilitates normalization of blood pH. Thus, balanced solutions are considered less likely to exacerbate acid-base disturbances (13, 14).

Lactated solutions are widely used for resuscitation and rehydration, but some clinicians fear they could cause hyperlactatemia or exacerbate lactic acidosis. Lactate is metabolized mainly in the liver, where it is oxidated to pyruvate by the cytosolic enzyme lactate dehydrogenase (LDH). Pyruvate can then be used to produce adenosine triphosphate (ATP) *via* the Krebs cycle, or it can be used for gluconeogenesis. Both processes consume hydrogen ions and lead to an excess of hydroxide ions, thus counteracting metabolic acidosis (2, 8, 15, 16). It has been shown that administration of large volumes of lactate-containing fluids leads to a transient elevation of plasma lactate. This is usually not associated with acidosis, because the lactate contained in the fluids is not lactic acid, but sodium-lactate, which is a salt and can be utilized for energy production (17–20). However, under anaerobic conditions, when tissues are forced to use glycolysis for energy production, pyruvate and hydrogen ions accumulate, which leads to a reversal of the reaction catalyzed by LDH, and lactate is produced. Additionally, hepatic clearance of lactate is impaired under hypoxic conditions (2, 21–23). Administration of lactate-containing solutions in patients with circulatory shock and patients with impaired liver function has been shown to lead to hyperlactatemia in several studies (18, 22, 24–26), for example in piglets with impaired liver function, where plasma lactate concentrations increased to over 10 mmol/L (26).

Acetate metabolism and its resulting increase in blood pH occurs faster than lactate metabolism, and additionally can take place in any cell able to perform aerobic oxygen metabolism (27). The highest metabolism rates are found in the cells of the skeletal muscle, heart, liver, and kidneys. Under aerobic conditions, acetate is metabolized to acetyl coenzyme A, and will then either enter the Krebs cycle or be consumed in gluconeogenesis, or lipogenesis. Both processes lead to consumption of protons, or formation of bicarbonate (27–29). Acetate-containing solutions have been associated with peripheral vasodilation, and reduced myocardial contractility in dogs and people, when used as a buffer for hemodialysis or hemofiltration (30–32).

Malate metabolism also occurs rapidly, as malate is a key intermediate in the Krebs cycle, and part of the aspartate-malate-shuttle, which transports electrons into the mitochondrial matrix. Thus, its metabolism not only leads to an increase in blood pH, but it is an alternative for energy production in both aerobic and anaerobic conditions (13, 33–37).

Veterinary studies comparing acetate- and lactate-containing solutions for resuscitation are scarce. A few studies in rats, pigs, and dogs compared different solutions with regards to mortality, hemodynamic effects, and plasma concentrations of electrolytes, lactate, glucose, and liver enzymes. Four of these studies were conducted in hemodynamically unstable patients (hemorrhagic shock, hemorrhagic shock with additional liver resection, and endotoxemia) (24, 38–40). In two of these, higher plasma lactate was found in patients after receiving a lactate-containing solution, compared with patients receiving a lactate-free solution (24, 38). Only one study found an advantage of using lactate-free solutions regarding improvement of metabolic acidosis (24). Three other studies were conducted in hemodynamically stable patients (dehydrated dogs, healthy pigs under anesthesia, and piglets with experimentally impaired liver function) (26, 41, 42). Patients receiving lactate-containing solutions showed higher plasma lactate, or reduced plasma lactate clearance, compared with patients receiving lactate-free solutions in all three studies. One of these studies showed improved acid-base status after administration of lactate-free solutions, compared with a lactated solution (26). In human medicine, studies and trials also show varying results (43). A randomized controlled trial in human burn patients found no significant difference in plasma lactate, acetate, or base excess, between patients receiving an acetate-containing solution, and patients receiving a lactate-containing solution (44). A human study in hemodynamically stable patients during elective surgeries found no difference between acetate- and lactate-containing solutions regarding acid-base status and inopressor requirements (45). In contrast to this, stable patients receiving acetate-containing solutions for more than 6 h intraoperatively showed higher lactate levels and progressive metabolic acidosis, compared to patients receiving lactate-containing solutions (46).

The aim of the present study was to compare short term effects of lactate- and acetate-containing replacement fluids on acid-base status, electrolytes, and plasma lactate, when given as a bolus to dogs with fluid deficits.

## Materials and methods

### Study population

This prospective, randomized, clinical study was approved by the Ethics Committee of the Center for Clinical Veterinary Medicine at the LMU Munich (reference number 153-09-12-2018). One hundred client-owned dogs presenting to the emergency service of the Clinic of Small Animal Medicine, LMU, Munich, Germany, were enrolled. Dogs were assigned to receive an intravenous fluid bolus by the attending clinician, based on patient history and physical examination (heart rate, pulse quality, mucous membrane color, capillary refill time, surface temperature of extremities, and level of mentation). Dogs were included in the study if the clinician decided to administer a fluid bolus, and owners gave oral consent. Exclusion criteria were age under 3 months, a history or clinical evidence of decreased liver function, a history or clinical evidence of cardiac disease potentially worsened through fluid bolus therapy, and severe electrolyte deviations before resuscitation (sodium <130 mmol/L; >160 mmol/L; potassium <3 mmol/L; >6 mmol/L). If a dog required more than one fluid bolus for cardiovascular stabilization, only the measurements and parameters before and after the first bolus were included in the data analyzed here. Dogs requiring resuscitation with hypertonic solutions or colloids were not included in the study. For each dog included in the study, the attending clinician documented an identification number, the type of fluid used, and the vital parameters before and after the fluid bolus.

### Interventions

All dogs were randomly assigned to receive either an acetate-containing solution (ACET; Sterofundin ISO^®^, B. Braun Vet Care, Tuttlingen, Germany) or a L-lactate-containing solution (LAC; Hartmann's Lactated Ringer's^®^, B. Braun Vet Care, Tuttlingen, Germany). Both solutions have different electrolyte concentrations. While Sterofundin ISO^®^ has higher sodium, chloride, and calcium concentrations, and additionally contains magnesium, Hartmann's Lactated Ringer's^®^ has a higher potassium concentration and does not contain magnesium (Table 1). Randomization was performed using an open-source program (https://www.randomizer.org). The respective solution was administered to each dog at 30 mL/kg body weight *via* an intravenous catheter over 10–15 min. Physical examination (heart rate, pulse quality, mucous membrane color, capillary refill time, surface temperature of extremities, and level of mentation) and venous blood gas analysis were performed directly before, and 10 min after bolus administration of the respective fluid in each dog.

**Table 1:** Electrolyte composition of the resuscitation fluids used in the study.

**Parameter**	**Sterofundin ISO** ^®^	**Hartmann's lactated Ringer's** ^®^
Sodium (mmol/L)	145.00	130.49
Chloride (mmol/L)	127	112
Potassium (mmol/L)	4.00	5.37
Calcium (mmol/L)	2.50	1.84
Magnesium (mmol/L)	1	0
Latate (mmol/L)	0	27.84
Acetate (mmol/L)	24	0
Malate (mmol/L)	5	0
Osmolarity (mosmol/L)	308	277
pH	5.1–5.9	5.0–7.0

### Blood collection and laboratory analysis

Blood collection was performed either *via* venipuncture, or *via* 3-syringe technique from an indwelling venous catheter from the cephalic, or the lateral saphenous vein. At least three times the catheter's priming volume was aspirated before sample collection. Commercially available, heparinized syringes were used for sample collection (Blutgas Monovette, Sarstedt AG & Co. KG, Nümbrecht, Germany). Sample analysis was performed immediately after anaerobic collection (Siemens RAPIDpoint^®^ 500 System, Siemens Healthcare GmbH, Erlangen, Germany), and the temperature was corrected to the patient's body temperature. Analyzed parameters included cpH (temperature corrected blood pH), cpCO_2_ (temperature corrected partial pressure of venous carbon dioxide), bicarbonate, base excess, anion gap, lactate, sodium, chloride, and potassium.

### Statistical analysis

Power analysis was performed with an open source software (powerandsamplesize.com). To detect a sodium difference of 3 mmol/L with a standard deviation of 3 mmol/l, with a power of 0.8, and an alpha error of 5%, 44 dogs per group were required.

For statistical analysis, a commercial software (Prism 5 for Windows, Graph Pad Software, Inc. San Diego, USA) was used. Normality was analyzed with omnibus D'Agostino K2 test. Non-normally distributed data are presented as median (minimum–maximum), normally distributed data are presented as mean ± standard deviation. Median age and mean weight of dogs in the ACET and the LAC group were compared using Mann-Whitney-U test and *t*-test, respectively. A Wilcoxon-matched-pairs signed rank test was used to compare plasma electrolyte concentrations, plasma lactate, and venous blood gas values in both groups before and after fluid resuscitation. Changes of these parameters between the two groups were compared depending on normality with a *t*-test, or a Mann-Whitney-U test. Numbers of patients with categorized physical parameters were analyzed using Chi-square test. A *p-*value ≤ 0.05 was considered significant.

## Results

### Study population

One hundred dogs were included in this study, and five were excluded due to extreme plasma sodium or potassium values. Male dogs were overrepresented (60/95) over females (35/95). The median age and mean weight of 47 dogs in ACET were 8.0 years (0.3–16.0 years), and 21.8 ± 13.4 kg, respectively. In the LAC group (*n* = 48), median age and mean weight were 8.5 years (10.0–15.0 years), and 21.3 ± 12.9 kg, respectively. These were not statistically different in age (*p* = 0.328), or weight (*p* = 0.847). The most frequently presented breeds were Labrador Retriever (11/95), Golden Retriever (5/95), Jack Russel Terrier (5/95), Australian Shepherd (3/95), and Pug (2/95). Most dogs, however, were mixed breed (21/95).

### Physical examination

At presentation, a total of 75/95 dogs were tachycardic (heart rate ≥ 120 beats per minute (bpm); Table 2). The median heart rate before the fluid bolus was 132 (80–200) bpm in the ACET group and 140 (80–200) bpm in the LAC group. In both groups, the heart rates improved significantly with fluid administration (see Supplementary material). The median heart rate decreased to 120 (72–160) bpm in ACET (*p* = 0.012), and to 120 (72–200) bpm in LAC (*p* = 0.012; Table 3). There was no significant difference between the two groups comparing the magnitude of the decrease in heart rate (*p* = 0.737; Table 4). The capillary refill times (CRTs) before fluid administration were prolonged (≥ 2 s) in 19/95 dogs (ACET: *n* = 7; LAC: *n* = 11), while 6/95 dogs had a prolonged CRT (ACET: *n* = 1; LAC: *n* = 5) after the bolus. After fluid therapy, significantly more dogs had a normal CRT (ACET: *p* = 0.012; LAC: *p* = 0.017). The subjective pulse quality was reduced in 42/95 dogs before bolus administration (ACET: *n* = 19; LAC: *n* = 23), and in 21/195 dogs afterwards (ACET: *n* = 10, *p* = 0.014; LAC: *n* = 10, *p* = 0.009). The mucous membrane color was found to be pale (*n* = 11), pink (*n* = 34), or hyperemic (*n* = 50) before fluid therapy, and normalized significantly after the bolus (pale *n* = 4, pink *n* = 80, hyperemic *n* = 11; *p* < 0.001 in ACET and LAC; Table 2).

**Table 2:** Physical examination parameters of 95 dogs presenting to the emergency service with signs of fluid deficit, measured before and after an intravenous fluid bolus of 30 mL/kg of either an acetate-containing (ACET), or a lactate-containing (LAC) fluid.

**Parameter**	**Before fluid bolus**		**After fluid bolus**		***p*** **-value**	
	**ACET (*****n** **=*** **47)**	**LAC (*****n** **=*** **48)**	**ACET (*****n** **=*** **47)**	**LAC (*****n** **=*** **48)**	**ACET**	**LAC**
Heart rate					0.121	0.339
<120 bpm	11	9	19	14		
≥120 bpm	36	39	28	34		
Capillary refill time					0.012	0.017
Normal (1–2 s)	23	19	37	34		
Prolonged (>2 s)	7	11	1	5		
Shortened (<1 s)	13	13	8	7		
Not measured	4	5	1	2		
Pulse quality					0.014	0.009
Normal	21	19	35	34		
Reduced	19	23	10	11		
Hyperdynamic	7	6	2	3		
Mucous membrane color					<0.001	<0.001
Normal (pink)	20	14	39	41		
Pale	4	7	2	2		
Hyperemic	23	27	6	5		

*The numbers of dogs in each group before and after fluid therapy were compared using chi-square test*.

**Table 3:** Heart rate, venous blood gas values, serum lactate and serum electrolyte concentrations before (pre) and after (post) resuscitation with either an acetate-containing (ACET), or a lactate-containing (LAC) fluid in 95 dogs with fluid deficits, presented as median and range.

**Parameter**	**ACET (*****n** =* **47)**			**LAC (*****n** =* **48)**		
	**pre**	**post**	**p**	**pre**	**post**	**p**
Heart rate (/min)	132 (80–200)	120 (72–160)	0.019*	140 (80–200)	120 (72–200)	0.012*
pH	7.34 (7.14–7.54)	7.34 (7.11–7.44)	0.804	7.32 (7.10 −7.46)	7.34 (7.14–7.42)	0.206
cpCO_2_ (mmHg)	40.8 (15.3–54.4)	39.8 (27.6–49.4)	0.751	40.3 (20.8–64.7)	37.9 (22.2–63.8)	0.203
Bicarbonate (mmol/L)	20.5 (7.4–29.2)	19.4 (7.3–28.1)	0.548	20.7 (7.6–26.9)	19.8 (8.6–26.2)	0.289
Base excess (mmol/L)	−4.4 (-18.9–11.2)	−6.5 (21.0–5.4)	0.333	−4.3 (-23.5–6.8)	−5.9 (-21.3–3.8)	0.266
Anion gap (mmol/L)	16.2 (5.0–26.6)	13.3 (3.3–22.2)	<0.001*	17.3 (8.4–36.4)	14.6 (7.1–27.7)	<0.001*
Lactate (mmol/L)	2.4 (1.0–8.6)	1.8 (0.7–8.5)	0.016*	2.6 (1.0–12.9)	3.2 (0.6–10.6)	0.169
Sodium (mmol/L)	142.9 (134.3–152.8)	142.7 (133.8–152.7)	0.555	143.4 (131.5–151.1)	142.3 (128.3–158.8)	0.016*
Potassium (mmol/L)	4.1 (3.1–5.1)	3.7 (2.7–4.9)	<0.001*	4.0 (3.1–5.8)	3.9 (2.9–5.5)	0.001*
Chloride (mmol/L)	111.0 (96.0–123.0)	114.0 (100.0–121.0)	<0.001*	109.0 (95.0–124.0)	111.0 (99.0–127.0)	<0.001*
Ionized calcium (mmol/L)	1.26 (1.02–1.74)	1.29 (0.99–1.69)	0.014*	1.26 (1.13–1.35)	1.25 (1.07–1.37)	0.186

The median values within each group (ACET and LAC) were compared before and after fluid therapy using Wilcoxon-matched-pairs signed rank test. ^*^ signifies p-values ≤ 0.05.

**Table 4:** Changes in heart rate, venous blood gas values, serum lactate, and serum electrolyte concentrations after an intravenous fluid bolus with an acetate-containing (ACET) vs. a lactate-containing (LAC) fluid in 95 dogs with fluid deficits.

**Changed parameter after fluid bolus therapy**	**ACET**	**LAC**	**xsp**
Heart rate (/min)	−10 ± 27	−12 ± 30	0.737
pH	−0.01 (−0.23–0.09)	0.01 (−0.10–0.14)	0.269
cpCO_2_ (mmHg)	−0.1 ± 6.6	1.5 ± 7.9	0.268
Bicarbonate (mmol/L)	−0.2 ± 1.8	−0.3 ± 1.5	0.733
Base excess (mmol/L)	−0.4 (−19.3–8.4)	0.0 (−8.2–4.9)	0.826
Anion gap (mmol/L)	−3.5 ± 3.3	−2.4 ± 3.9	0.157
Lactate (mmol/L)	−0.3 (−3.6–4.4)	0.42 (−5.91–2.94)	0.015*
Sodium (mmol/L)	−0.2 (−11.2–8.3)	−1.5 (−6.9–13.5)	0.039*
Potassium (mmol/L)	−0.4 (−1.0–0.5)	−0.2 (−1.5–0.9)	0.006*
Chloride (mmol/L)	3.0 (−3.0–8.0)	1.5 (−5.0–14.0)	<0.001*
Ionized calcium (mmol/L)	0.02 (−0.21–0.30)	−0.01 (−0.19–0.11)	0.016*

The numerical differences between pre- and post-resuscitation values are presented as mean and standard deviation, if normally distributed, and as median and range, if not normally distributed. Mean values were compared using t test, median values were compared using Mann-Whitney-U test. ^*^ signifies p-values ≤ 0.05.

### Venous plasma lactate levels

A total of 62/95 dogs had elevated (>2.0 mmol/L) plasma lactate levels at presentation (ACET: *n* = 28; LAC: *n* = 34). The median plasma lactate before fluid therapy was 2.4 mmol/L (1.0–8.6 mmol/L) in the ACET group, and 2.6 mmol/L (1.0–12.9 mmol/L) in the LAC group. After fluid therapy, there were less dogs in ACET with increased plasma lactate (*n* = 20), but in LAC, their number had increased (*n* = 40). The median plasma lactate concentration after fluid therapy was 1.8 (0.7–8.5) mmol/L in ACET (*p* = 0.016), and 3.2 (0.6–10.6) mmol/L in LAC (*p* = 0.169; Table 3, Supplementary material). Plasma lactate concentrations were significantly higher in LAC than in ACET after fluid administration (*p* = 0.015; Table 4).

### Venous acid-base parameters

Most dogs (78/95) showed acid-base disorders before therapy (metabolic acidosis: *n* = 70; respiratory acidosis: *n* = 2; mixed metabolic and respiratory acidosis: *n* = 2; metabolic alkalosis: *n* = 2; and respiratory alkalosis: *n* = 3). After fluid administration, 74/95 dogs had acid-base disturbances (metabolic acidosis: *n* = 66; respiratory acidosis: *n* = 2; mixed metabolic and respiratory acidosis: *n* = 4; metabolic alkalosis: *n* = 1; and mixed acidotic and alkalotic disorders: *n* = 1; Table 5). When looking at the acid-base parameters individually, only the anion gap changed significantly with fluid therapy. It decreased from a median of 16.2 mmol/L (5.0–26.6 mmol/L) in ACET, and 17.3 mmol/L (8.4–36.4 mmol/L) in LAC to 13.3 mmol/L (3.3–22.2 mmol/L) in ACET (*p* < 0.001) and 14.6 mmol/L (7.1–27.7 mmol/L) in LAC (*p* < 0.001; Table 3). All other individual acid-base parameters did not show any significant changes after fluid administration, or between the two fluid types (Tables 3, 4).

**Table 5:** Acid-base status of 95 dogs presenting to the emergency service with signs of fluid deficit, before and after an intravenous fluid bolus of 30 mL/kg of either an acetate-containing (ACET), or a lactate-containing (LAC) fluid.

**Acid-base status**	**Before fluid bolus**		**After fluid bolus**	
	**ACET (*****n** =* **47)**	**LAC (*****n** =* **48)**	**ACET (*****n** =* **47)**	**LAC (*****n** =* **48)**
Metabolic acidosis	35	35	37	29
Respiratory acidosis	1	1	0	2
Mixed acidosis	1	1	0	4
Metabolic alkalosis	2	0	1	0
Respiratory alkalosis	2	1	0	0
Mixed disorder: respiratory acidosis and metabolic alkalosis	0	0	0	0
Mixed disorder: respiratory alkalosis and metabolic acidosis	0	0	0	1
Normal acid-base parameters	7	10	9	12

*Categorization into acid-base disturbances was performed retrospectively, according to the following blood gas parameters: pH (reference range 7.29–7.45), temperature corrected partial pressure of carbon dioxide (cpCO_2_; reference range 27–51 mmHg), bicarbonate (HCO_3_; reference range 18–25 mmol/L)*.

### Venous electrolyte concentrations

In both groups, plasma potassium decreased significantly after fluid administration (ACET: *p* < 0.001; LAC: *p* = 0.001; Table 3). This effect was more pronounced in dogs receiving the lactate-containing solution (*p* = 0.006; Table 4). Plasma chloride increased with both solutions (*p* < 0.001 in ACET and LAC; Table 3, Supplementary material). The increase was significantly stronger in the ACET group (*p* < 0.001; Table 4). Plasma sodium was significantly reduced in LAC after fluid therapy (*p* = 0.016) but showed no significant change in ACET (*p* = 0.555; Table 3). Ionized calcium concentrations increased significantly in ACET (*p* = 0.014) but decreased insignificantly in LAC (*p* = 0.186; Table 3).

## Discussion

In the present study, two different crystalloid solutions were compared regarding their effects on acid-base status, electrolytes, and plasma lactate concentrations, when administered as an intravenous bolus of 30 mL/kg to dogs without severe electrolyte deviations. Both solutions had only minor influence on acid-base status, but plasma lactate concentrations were reduced more effectively in the ACET group. There were no clinically relevant changes in plasma electrolyte concentrations, although the two solutions had different influences on plasma potassium and chloride.

All dogs underwent physical examination at presentation. The majority of dogs presented with mild tachycardia (75/95), abnormal pulse quality (55/95), and abnormal mucous membrane color (61/95). The CRT was not measurable in nine dogs due to either pallor, or uncooperative behavior, but there were more dogs with an abnormal CRT (44/95), then with normal CRT (43/95). However, there were still many dogs with one or more normal physical examination parameters, indicating that most dogs included in this study did not have severe fluid deficits, and were not in severe shock. Nevertheless, there was an apparent response to fluid therapy. The median heart rate was significantly reduced in both groups, and the numbers of dogs with abnormal CRT, pulse quality and mucous membrane color also significantly declined. Dogs receiving the acetate-containing solution did not show signs of hypotension, or cardiovascular deterioration, so this possible adverse effect of acetate has not been noted in this study. However, without blood pressure measurements, mild changes in vascular tone cannot be fully excluded. Both fluids seem to perform equally well in improving the cardiovascular status, without significant difference between the two groups regarding reduction of median heart rate. As both fluids are isotonic crystalloids, and were administered at the same volume per body weight, they were expected to have a similar effect on intravascular volume and preload, and to distribute similarly in the extracellular space (3).

Most dogs (62/95) had elevated plasma lactate levels at presentation, which most likely indicates anaerobic metabolism. Metabolic acidosis with hyperlactatemia is a common consequence of tissue hypoxia, although tissue oxygen supply depends on additional factors, such as vascular tone, regional blood flow, hemoglobin concentrations, and partial pressure of oxygen (16, 21). While dogs in ACET showed a significant decrease of plasma lactate after fluid administration, dogs in LAC showed no significant changes. The decrease in the ACET group indicates improved blood circulation and tissue oxygenation, which is corroborated by the improvement of physical examination parameters. The lack of change in the LAC group can be contributed to the short time span between fluid administration and plasma lactate measurement. Crystalloids are expected to distribute within the extracellular compartment within 30 min after intravenous administration (1), and the half-life of plasma lactate ranges from 20–60 min in healthy adult people (47, 48), as well as in healthy dogs (49–52). In the present study, plasma lactate was measured 10 min after fluid administration, which is likely not enough time for full metabolism.

When looking at the existing literature, the effects of lactate-containing solutions on plasma lactate levels vary. A review and meta-analysis of 29 studies comparing the effects of acetate- vs. lactate-containing solutions in people found increased plasma lactate levels during or after administration of lactate-containing solutions in 14 studies, while five studies found no significant difference between groups (43). In a study in people, lactated Ringer's (LR) was compared to normal saline (NS), when administered as an intravenous bolus of 30 mL/kg to healthy patients. Plasma lactate was measured directly before, and 5 min after conclusion of bolus administration, but the administration lasted 47 min on average. No significant difference between the plasma lactate increase in the NS group, and the increase in the LR group was found (19). A similar study with 24 healthy adults receiving 1 L of either lactated Ringer's or saline over the course of 1 hour also did not observe a difference in plasma lactate levels between the groups. Plasma lactate was measured before, during, and up to 240 min after bolus administration (53). These two studies used fluid volumes similar to the one in the present study, but measurements of plasma lactate were done after a much longer time span. Another study evaluated plasma lactate levels in healthy dogs after administration of 180 mL/kg/h for 1 h. The dogs' plasma lactate levels increased within the first 10 min, but returned to baseline after 1 h (17). In another study, dehydrated dogs were resuscitated with either a lactate-containing, or an acetate-containing solution. The total fluid volume administered depended on the individual patients' requirements, but the median volume was > 100 mL/kg, administered over 6 to 24 h. Plasma lactate levels decreased significantly in both groups, but the decrease was more pronounced in the acetate-group (41). A study in people undergoing major surgeries found higher intraoperative lactate levels in patients receiving 10 mL/kg/h of a lactate-containing solution, compared with the same rate of an acetate-containing solution. Measurements were done every 2 h from baseline to 8 h after the start of infusion (54). These studies also measured the patients' plasma lactate after more than 30 min but used a much greater fluid volume than in the present study, which could explain the greater impact on plasma lactate levels.

All dogs in the present study showed signs of fluid deficits, and 70/95 dogs presented with metabolic acidosis. Fluid deficits can lead to hypoperfusion, and hypoxic tissues utilize anaerobic glycolysis to produce energy, which leads to an increased production of hydrogen ions and lactate (2, 16). Thus, with pronounced fluid deficits, metabolic acidosis and hyperlactatemia are expected findings. After fluid therapy, there were still 66/95 dogs with metabolic acidosis, which is a rather low response. The changes in pH, cpCO_2_, bicarbonate and base excess after fluid therapy were not significant, and there was no difference between the two fluids regarding their effects on acid-base parameters. One possible explanation for this is the fact that the second blood gas sample was taken only 10 min after bolus administration, as this is likely not enough time for full distribution and metabolism (1). However, other mechanisms, such as underlying conditions or co-morbidities, influence the acid-base status as well, and can exacerbate metabolic acidosis, or lead to different forms of acid-base disturbances (mixed acid-base disturbances, alkalotic changes, and/or respiratory acid-base disorders) (3). Overall, both fluid types seem to have a similarly low impact on the patients' acid-base status, when given as an intravenous bolus of 30 mL/kg. This is in accordance with another study in dehydrated dogs, which also found no significant difference in acid-base parameters between the two fluids, when administered over a period of 6 to 24 h for rehydration (41). According to a meta-analysis of 29 studies comparing acetate- and lactate-containing fluids, the results concerning the acid-base status were conflicting. Thirteen studies found no differences between the groups, two studies found a lower blood pH in the lactate-group, two studies found a lower blood pH in the acetate-group, and four studies found no significant difference, but a transiently elevated base excess in the acetate-group (43).

The anion gap decreased significantly after fluid resuscitation with both solutions. This arithmetic value represents the difference between unmeasured anions and unmeasured cations. An actual anion gap, however, does not exist, as electroneutrality is maintained at all times (55). In healthy dogs, the calculated anion gap ranges from 12 to 24 mmol/L (3). An increased anion gap is usually attributed to an increase in unmeasured anions, e.g., lactate, phosphate, or ketoacids (3, 10, 55). While there was an overall decrease in anion gap after fluid administration (77/95), only 11/95 dogs had an anion gap of more than 24 mmol/L to start with. Most dogs (71/95) showed an anion gap within the reference range, and 13/95 already had a decreased anion gap at presentation. Thus, the decrease of the anion gap after fluid resuscitation is likely to be attributed to dilution of electrolytes, plasma proteins, and their net negative charge (3).

There were no clinically relevant changes in plasma electrolyte concentrations after fluid resuscitation in both groups. Both fluids used in this study were balanced crystalloids with electrolyte concentrations similar to the patients' plasma, so clinically relevant electrolyte deviations were not expected. However, plasma chloride concentrations increased significantly more in the ACET group, possibly reflecting the higher chloride concentration in Sterofundin ISO^®^ compared to Hartmann's Lactated Ringer's^®^. The former solution also has a higher sodium concentration compared to lactated Ringer's, but did not increase plasma sodium concentrations, although the general decrease of plasma sodium in both groups after bolus administration was less pronounced in the ACET group. Increases in plasma chloride but not in plasma sodium have been reported when isotonic saline was used for resuscitation (3, 8, 9, 56). The pathophysiologic explanation is that the chloride concentration of saline is higher than that of the extracellular fluids, which leads to the displacement of bicarbonate with chloride and causes hyperchloremic metabolic acidosis. At the same time, the intravascular volume expansion leads to an increased renal perfusion which suppresses plasma renin activity and increases natriuresis (3, 8, 57). Sterofundin ISO^®^ does indeed contain a slightly supraphysiological chloride concentration of 127 mmol/L. To the authors' knowledge, acetate-containing solutions have not been reported to cause an increase in plasma chloride. A study with 40 healthy dogs receiving either saline, saline with dextrose, or Ringer's lactate, reported an increase of plasma chloride in all dogs except the ones that received the acetate-containing solutions. The measurements were performed after 1 h of infusion, and again 1 and 3 h after infusion was completed, so the timing could have been an important factor (58). The most likely reason for the stronger increase in plasma chloride in the ACET group seems to be a short-term effect of the higher chloride concentration of Sterofundin ISO^®^. The stronger decrease in plasma sodium in the LAC group can also be attributed to the lower sodium concentration and *in vivo* osmolality of Ringer's Lactate (7, 18).

In both groups, a significant reduction of plasma potassium was found after fluid therapy. Potassium is the main intracellular cation and highly important for the heart and skeletal muscles, nerve conduction, and renal function. Its balance between the extracellular and the intracellular space is influenced by aldosterone, insulin, β-adrenergic agonists, and bicarbonate (3, 59). Supplementation of balanced replacement fluids (such as the ones used in this study) with potassium is recommended for long-term fluid therapy to avoid iatrogenic hypokalemia (3, 60). In the present study, fluids were only given as a bolus and clinically significant potassium decrease was not expected with this treatment. None of the dogs in this population showed clinically relevant potassium changes after fluid resuscitation.

Plasma ionized calcium concentrations were influenced differently by the solutions, with a significant, but clinically irrelevant increase in the ACET group, and a minor decrease in the LAC group. This also correlates with the calcium concentrations of the respective fluids. Ionized calcium is the biologically active calcium fraction in the plasma, while protein-bound calcium is thought to be biologically inactive and merely a storage pool or buffering system for the ionized calcium (3). The protein-binding is pH-dependent, so that a higher plasma pH leads to stronger protein-binding and a lower pH to increased ionized calcium (61). Both fluids only had minor influence on plasma pH, therefore it can be assumed that their influence on plasma calcium was not mediated via their influence on plasma pH.

Overall, the electrolyte concentrations of the two fluids only had minor, and most likely clinically irrelevant influence on the patients' electrolyte levels. However, for patients with significant electrolyte deviations, this situation might be different. Further studies are needed to evaluate the impact of different fluids and their different electrolyte concentrations on patients with significant electrolyte deviations.

There are several limitations to this study. The main criterion for inclusion was the clinician's assessment of the cardiovascular status of the dogs. This has led to the inclusion of a very heterogeneous group of dogs with a wide variety of underlying diseases, affecting different organ systems, including the respiratory tract and the kidneys. Responses to fluid therapy can differ depending on the underlying problem, and disturbances of different organ systems influence the acid-base status and its regulation (3). A larger study population could have facilitated more significant findings and differences between the two fluid types.

None of the dogs were assessed for fluid responsiveness before inclusion in the study, they were included upon physical examination parameters only. As most dogs only had mild fluid deficits, the findings for the present study might not be representative for dogs with severe fluid losses or shock. Blood pressure measurements would have been a valuable addition in assessing the cardiovascular status of the dogs, and for assessing adverse effects of acetate, but were not performed in most dogs and thus could not be included in the analysis. Liver function was not assessed *via* measurement of ammonia or bile acids, which leaves a possibility for inclusion of dogs with non-clinically apparent liver dysfunction. Also, no cardiac ultrasound was performed before inclusion, so mild cardiac disease in some patients cannot be fully excluded.

The fluid volume administered in the present study was comparatively small, a larger influence on acid-base status and/or electrolytes could be found when larger volumes are administered. The short time span between fluid administration and the second measurement of venous blood gas parameters also limits the information of the study. A third measurement after 30 min or more could have revealed which of the findings are only transient effects, and which are still significant after enough time for distribution and metabolism. Lastly, the physical examinations before and after the fluid bolus were performed by the emergency clinician on duty at the time of presentation. Some examiner-dependent variation of subjective parameters cannot be fully excluded.

In conclusion, both fluids performed well in improving the cardiovascular status of dogs with signs of fluid deficits, when given as a resuscitation bolus of 30 mL/kg. Administration of an acetate-containing solution resulted in lower plasma lactate levels, compared with lactate-containing solutions. Both solutions only had minor influence on acid-base status. There were differences between the two fluids regarding their influence on plasma electrolytes, but none of these changes were clinically relevant.

## Data availability statement

The raw data supporting the conclusions of this article will be made available by the authors, without undue reservation.

## Ethics statement

The animal study was reviewed and approved by Ethics Committee of the Center for Clinical Veterinary Medicine at the LMU Munich. Written informed consent for participation was not obtained from the owners because both treatment solutions are licensed for dogs, and were administered based on the patients' fluid deficit and clinical signs.

## Author contributions

RD, UK-R, and AH: conceptualization, investigation, and methodology. UK-R and AH: data curation. RD and UK-R: formal analysis. KH and RD: funding acquisition and resources. RD: supervision. UK-R: writing—original draft. AH, KH, and RD: writing—review and editing. All authors contributed to the article and approved the submitted version.

## Funding

B. Braun sponsored the residency position of UK-R. They did not financially support the project/study.

## Conflict of interest

Author AH was employed by AniCura Tierklinik Haar GmbH. The remaining authors declare that the research was conducted in the absence of any commercial or financial relationships that could be construed as a potential conflict of interest.

## Publisher's note

All claims expressed in this article are solely those of the authors and do not necessarily represent those of their affiliated organizations, or those of the publisher, the editors and the reviewers. Any product that may be evaluated in this article, or claim that may be made by its manufacturer, is not guaranteed or endorsed by the publisher.
