# Supplementary material for: Influence of acetate- vs. lactate-containing fluid bolus therapy on acid-base status, electrolytes, and plasma lactate in dogs
Source: Front Vet Sci. 2022 Jul 29;9:903091. doi: 10.3389/fvets.2022.903091 (PMC9372486; doi:10.3389/fvets.2022.903091)
Supplement: Supplementary file 1 [file Presentation_1.PPTX]

## Slide 1
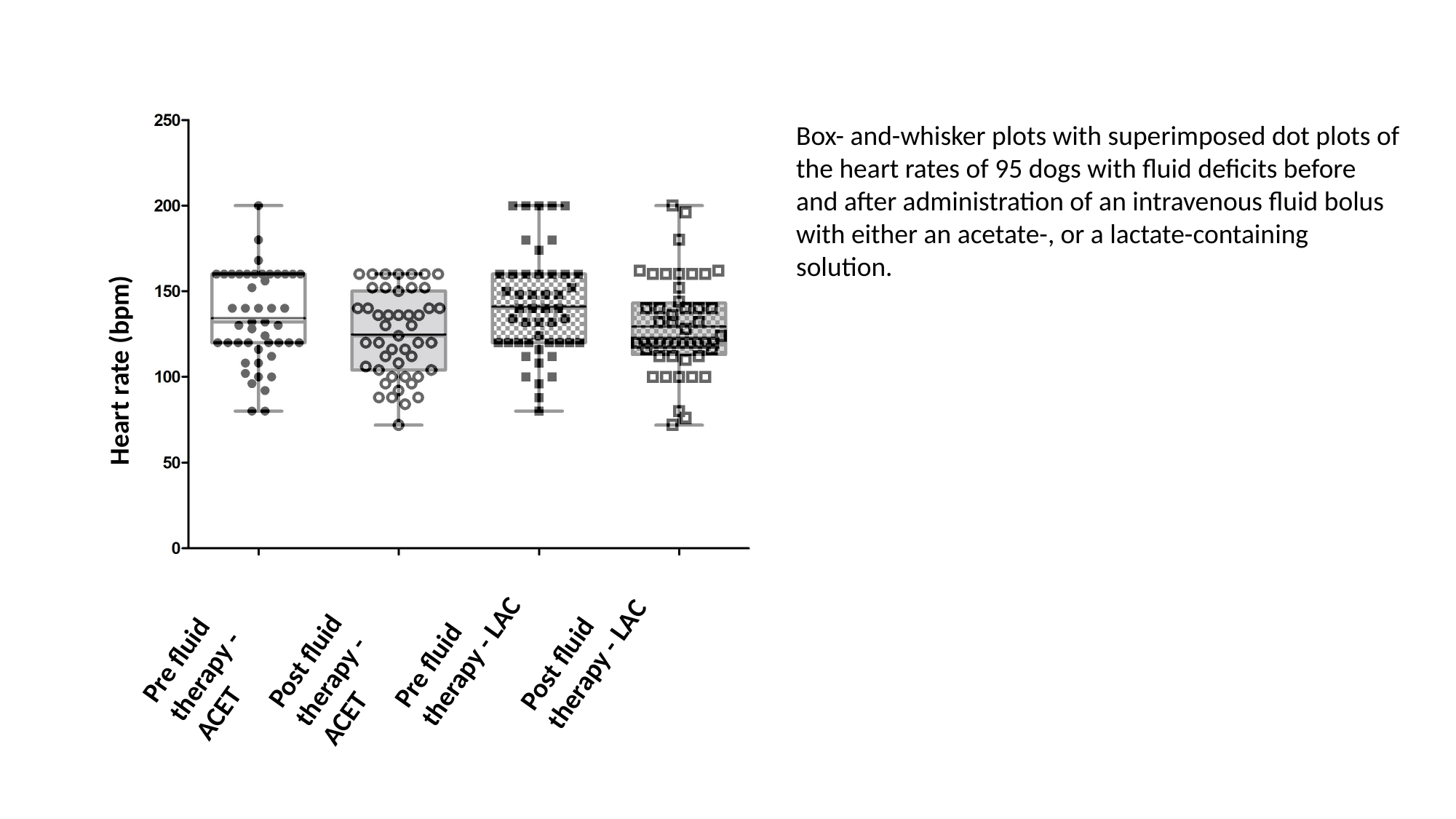

Box- and-whisker plots with superimposed dot plots of the heart rates of 95 dogs with fluid deficits before and after administration of an intravenous fluid bolus with either an acetate-, or a lactate-containing solution.
Heart rate (bpm)
Pre fluid therapy - ACET
Pre fluid therapy - LAC
Post fluid therapy - ACET
Post fluid therapy - LAC

## Slide 2
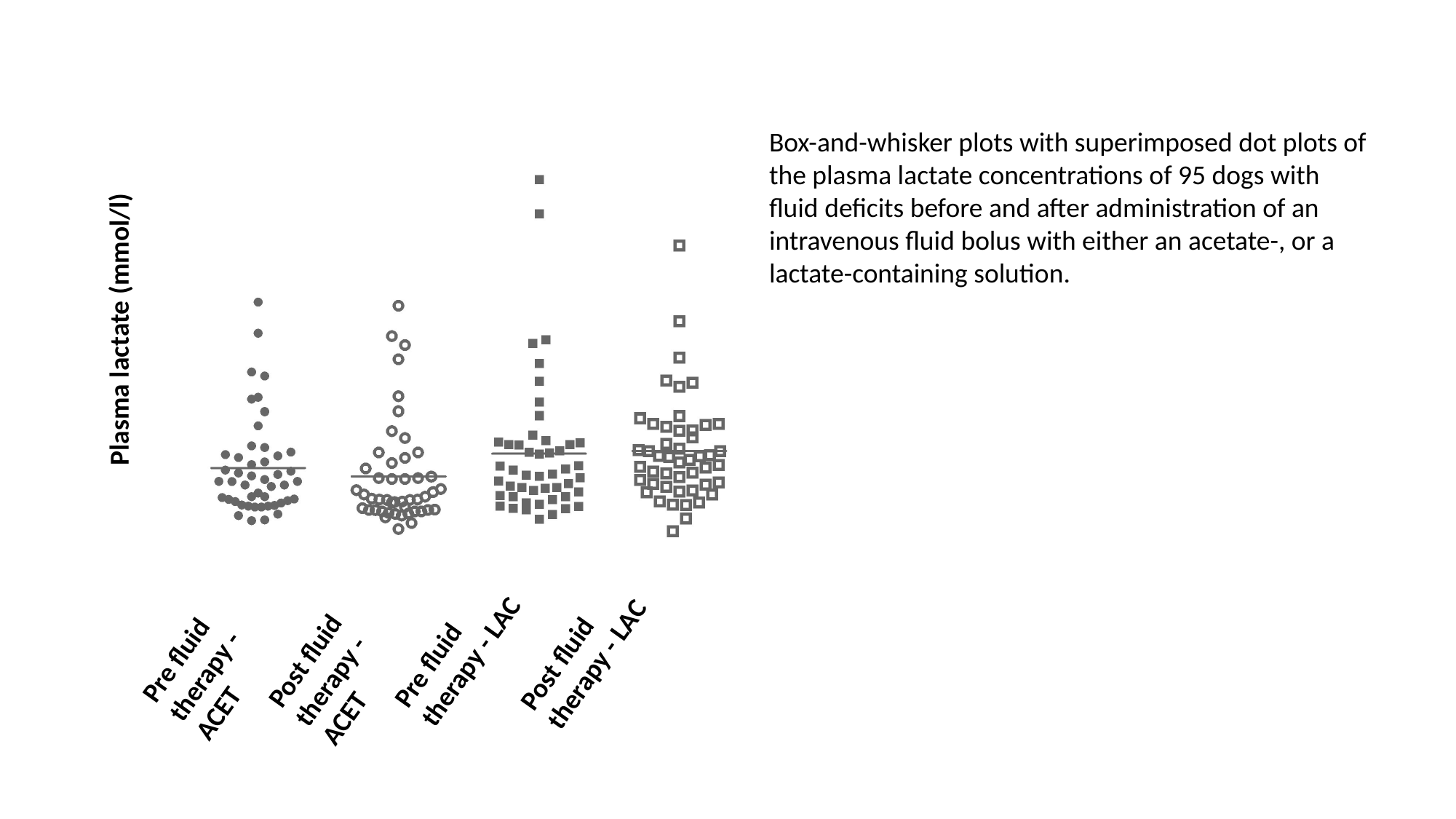

Box-and-whisker plots with superimposed dot plots of the plasma lactate concentrations of 95 dogs with fluid deficits before and after administration of an intravenous fluid bolus with either an acetate-, or a lactate-containing solution.
Plasma lactate (mmol/l)
Pre fluid therapy - ACET
Pre fluid therapy - LAC
Post fluid therapy - ACET
Post fluid therapy - LAC

## Slide 3
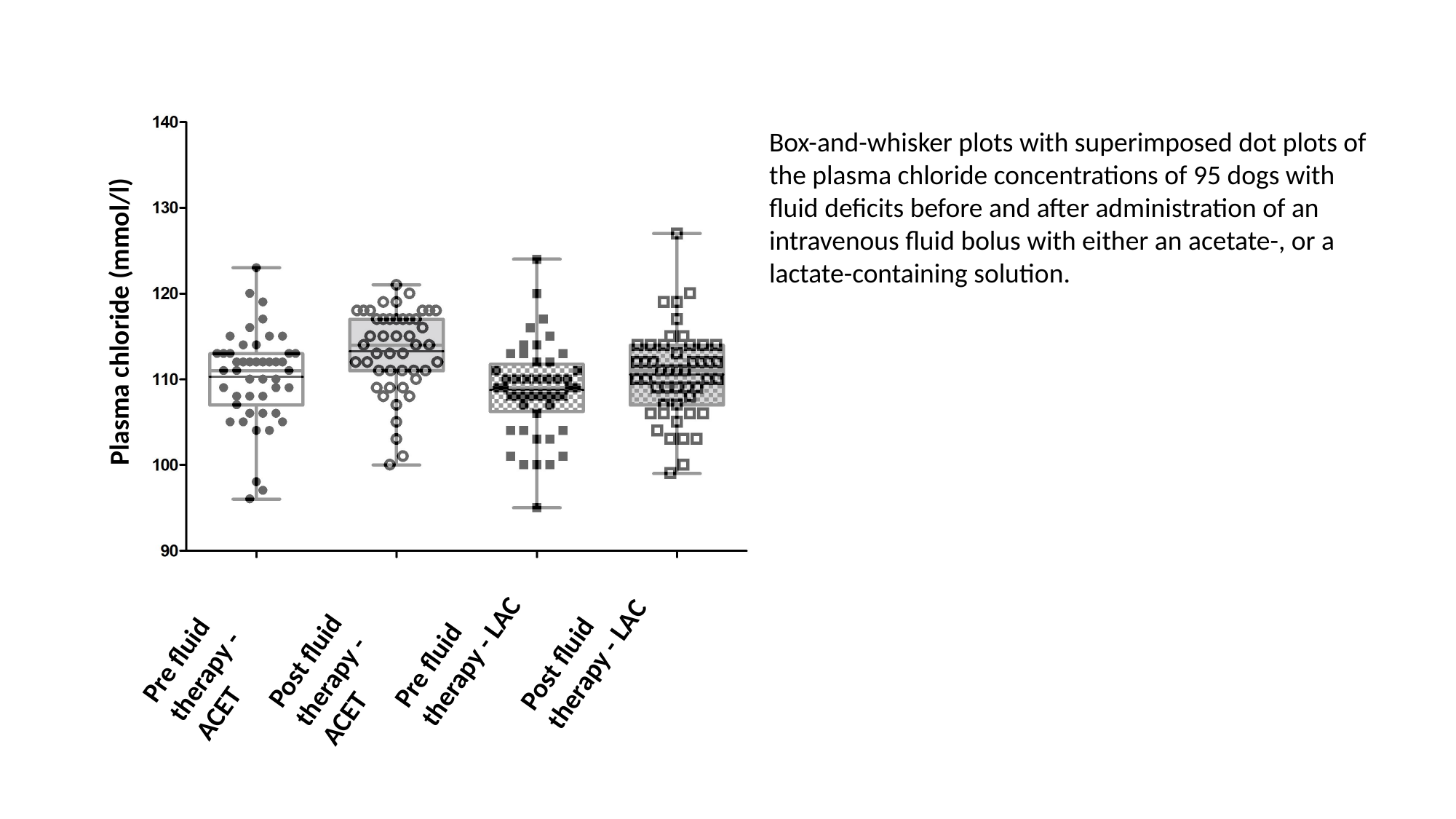

Box-and-whisker plots with superimposed dot plots of the plasma chloride concentrations of 95 dogs with fluid deficits before and after administration of an intravenous fluid bolus with either an acetate-, or a lactate-containing solution.
Plasma chloride (mmol/l)
Pre fluid therapy - ACET
Pre fluid therapy - LAC
Post fluid therapy - ACET
Post fluid therapy - LAC
